# Uncertainty and Demand for Insurance: A Theoretical Model of How Self-Control Manages the Optimal Decision-Making

**DOI:** 10.3389/fpsyg.2021.700289

**Published:** 2021-08-17

**Authors:** Mattia Adamo, Andrea P. Malizia

**Affiliations:** ^1^Laboratory for the Analysis of CompleX Economic Systems (AXES), IMT School for Advanced Studies Lucca, Lucca, Italy; ^2^Molecular Mind Laboratory (MoMiLab), IMT School for Advanced Studies Lucca, Lucca, Italy

**Keywords:** self-control, willpower, decision-making, consumer, utility, insurance

## Abstract

With the present work, we aim to mark a beginning line on the study of decision-making of potential consumers in the insurance sector, with the long-term purpose of defining the optimal cognitive processes to be undertaken when deciding whether to purchase insurance or not. Decision-making in conditions of uncertainty is influenced by the dual-self model doers/planner integrated with the hot–cold states and prospect utility function. Thus, we present a theoretical model of choice-making to evaluate the level of optimal self-control necessary to be exerted if the individual is either in the hot or in the cold state depending on the arousal. This theoretical choice-making model lays the ground for the decision journey by following the long-term utility and avoiding gross mistakes that could lead the consumer not to insure, when the odds suggest doing it, or vice versa, in situations when it would not be necessary.

## Introduction

In the insurance sector, decision-making processes of consumers are configured as choices in conditions of risk and uncertainty. The insurance firm is responsible for the risk underwriting, calculating a premium to be paid by the customer for the insurance purchase based on a set of factors deemed relevant at a large scale of customers, ensuring profitability. Uncertainty from the acquirer's view, the protagonist of the present article, is given by the subjectivity of each case (Ewald, 1991). It is expressed in terms of economic availability, risk disposition, trust in a competitive system of insurance (Abraham, 1985), among many other psychological factors, including the cognitive–affective interplay, that go far beyond the mere utility maximization (Kusev et al., 2017).

In a complex system with several correlating factors in dynamic conditions, insurance decision-making is still characterized by the impossibility of defining, *ex ante*, with confidence by the prospect acquirer, the probability of events in the future.

Against the desired mechanism of self-control triggered by a cognitive “controller” over an affective “controlee” on decision-making, Blanchette and Richards (2010) reviewed a series of articles to identify the association between affective states and cognitive mechanisms, hence on the subjectivity of the direction of self-control. Sometimes affective states hinder normatively correct thinking, while in other cases, they promote it (Gigerenzer and Gaissmaier, 2011). In a more practical description, tests can be made to assess how the combination between context, cognition, and emotions adds value to financial decision-making; in the hypothesis, this can be achieved when agents adaptively rely on heuristics in uncertain conditions (Forbes et al., 2015).

Indeed, emotions seem to prevail over more rational choices in financial and insurance decision-making when self-control is poorly executed due to either positive or negative affective states. Higgins (1997) was the first to describe the “regulatory focus,” attributable to self-control, based on a dual set of prevalent emotions either sought or avoided. In particular, promotion-focused individuals build their regulatory focus over the hope to match with the positive emotion toward their ideal self. Contrarily, prevention-focused individuals are concerned with avoiding the negative emotion and the relative outcome, hence taking their decisions in line with such a rationale, obeying their ought self. In either way, chronic promotion- or prevention-focused individuals' use of feelings to make a choice increased the monetary value of the chosen product (Avnet and Higgins, 2006). This is due to the hypothesis individuals decide and confirm their decision based on their orientation more than on an absolute idea of what is fair to do, re-directing them through regulatory fit (Avnet and Higgins, 2006). *De facto*, according to the regulatory fit theory, when people engage in decisions or choices with strategies that sustain their orientation, they “feel right” about what they are doing. This feeling-right experience consequently reduces the self-control mechanism, then transfers to subsequent evaluations. Hence, the feeling of what is right and what is wrong assumes a meaning relative to the goal itself, on the attitude toward the goal. This is seen as the modification effect on the perceived choice-value that people experience depending on the strategy used to evaluate the choice (Freitas and Higgins, 2002; Avnet and Higgins, 2003; Camacho et al., 2003).

## The Doers-Planner Model and its Role in Driving Dynamic Inconsistency

The economic literature has found in the doers-planner model and self-control one of the most influential systems in investigating uncertainty in decision-making (Thaler and Shefrin, 1981). In each moment, it is assumed that a planner and a doer are applying opposing forces in the agent. Suppose the prevalently rational cognitive state relatable to the planner represents altruistic time preferences, allowing the individual to maximize the long-run utility function. Contrarily, the emotionally affected cognitive state attributable to the doer, the actor of the decision-making instant, is myopic and selfish toward the costs and benefits of the living timeframe.

This has been widely adopted by many scholars who explicitly or implicitly recall Thaler's contrast between the myopic and instinctual system theorized as the doer and the deliberate, forward-looking system named as a planner. By attributing different names to the two selves, Benhabib and Bisin (2005) theorize a dualism between impulsive and controlled answers Loewenstein and O'Donoghue (2004) oppose effective processes with deliberative ones Fishbach et al. (2003) explain that individuals encounter temptation enticing them to stray from their chosen path and impeding progress toward goal attainment. These concepts give Thaler's idea regarding the relationship between the semi-autonomous selves and automatic and reflective systems. More specifically, Thaler highlights how the so-called planner “speaks” for the reflective system, following determined rules and acting in a controlled, rational, and deductive way, and how the doers are instead influenced by the fast, unconscious, impulsive, associative, and uncontrolled automatic system (Thaler and Sunstein, 2009). Fudenberg and Levine (2006, 2012) built a model opposing an impulsive short-run self to a patient and rational long-run self, by focusing on the ways through which the individual tries to control their impulsive and irrational answers (Gul and Pesendorfer, 2001, 2004a,b).

Such inability to correctly implement the optimal plan previously formulated is due to the existence of cognitive, emotional, social, cultural, and individual factors that mislead to confuse the perceived instinctual risks of the moment with the rational risks relative to the decision to be taken (Slovic et al., 1982; Douglas and Wildavsky, 1983; Kasperson et al., 1988; Wildavsky and Dake, 1990; Loewenstein et al., 2001; Joffe, 2003; Leiserowitz, 2006; Slovic, 2010; Brighetti et al., 2014; Outreville, 2014). This confusion would cause the overvaluation of short-term costs and benefits through a modification of the discount factor. In such moments, individuals are affected by loss-aversion, for which they weigh chances of loss much more than the chances of a gain of equivalent amount (Kahneman and Tversky, 1979, 2013).

This conflicting interplay has been investigated by many scholars on evaluating optimal alternatives, given costs and benefits distributed in different time horizons. *De facto*, the intertemporal choice is useful in explaining how the agent might find herself in the future when deciding whether to pursue insurance. Such choices assume considerable relevance in the lives of the individuals, contributing to determining the wealth of the entity (Frederick et al., 2002). Intertemporal decisions are supposed to be solved by selecting the optimal choice through time, thus, in the ideal model proposed by classic microeconomics, maximizing utility for the subject throughout the considered time frame. Recognized as one of the original references for intertemporal choices in classic microeconomics, Samuelson (1937) elaborated the discounted expected utility model.^1^ The agent does not evaluate the present and future consumption in absolute values, as the latter is supposed to be discounted exponentially by using a certain rate. The model elaborated by Samuelson represented dynamically coherent time preferences, identifying the human being as a perfectly rational agent. However, the same author raised concerns about applying the utility function outside the theoretical scope. Strotz (1955) first noted how the exponential discount function can only grant the property of coherence and that such coherence was not consistent among all individuals in all periods. Indeed, decision biases of individuals in intertemporal choice and choice under uncertainty have a common mechanism located in the utility function through time, probability, and payout dimensions (Chapman and Weber, 2006).

Several later empirical studies have highlighted how the characteristics of constancy and independence, proper of the individual discount factor, were systematically contradicted by the behavior of the actual agents. The resulting actions often showed incoherence—dynamic inconsistency—from the optimal choices as planned (Phelps and Pollak, 1968; Ainslie, 1975; Frederick et al., 2002). Thaler and Shefrin (1981) provided their first research in such a frame, where they recognized the evidence of Strotz and other scholars^2^ on dynamic inconsistency as a primary starting point. Strotz interpreted the phenomenon of dynamic incoherency in terms of changing tastes. Thaler and Shefrin (1981) explained such a conflictual divergence between plans and actual actions, conceptualizing a model in which the agent was animated by the presence of two sets of contradictory tastes in the same moment. Strotz's formulation hence remains in Thaler's approach. The innovation of the economic theory of self-control lies in the different time characteristics of the opponent and the conflict itself, which do not oppose today's preferences from those of tomorrow; but instead put two interacting forces, simultaneously present in the agent, in front of each other.

The authors also conducted their research starting with similar points relating self-control to the temptation problems arising at the origin of the individual's changes of preferences through time. They re-read the temptation-related problems offered by Gul and Pesendorfer (2001) in terms of intrapersonal conflict between deliberate and intuitive processes, explicitly recalling the assumptions made by Thaler and Shefrin (1981). By trying to explain the deviation from the optimal choices in dynamic inconsistency, in their early work, Barkan and Busemeyer (1999) reported the effect of experience on the reference point used for the evaluation of the decision problem. Other scholars even tried to focus on the causes of the change of preferences. O'Donoghue and Rabin (1998) assume that the agents are naïf, have present-oriented tastes but are unconscious of that. Laibson (1997) hypothesizes a model which sees the agents mainly as rational planners presenting problematic time preferences that might mislead them.

Despite the fundamental contributions and theoretical framing of several models herein discussed, to the best of the authors' knowledge, this research area lacks a united and shared mechanism that explains the exertion of the affective states on the doers/planner model in the insurance decision-making.

## How Arousal Modulates Hot and Cold States and Affects Self-Control in Dynamic Inconsistency

Far from being complete, applying the doers-planner model in insurance decision-making highlights the impossibility of linking the effect of self-control on arousal at different times. Loewenstein (1996) conceptualized hot and cold states as counterposed actors; he evidenced how emotional and irrational variables strongly influence the individual in certain situations as the hot state. Such variables have a decisive impact on decision processes that, differently from what happens in the cold states, are no more under the control of reason, or at least they are free from conscious rationalization. Bernheim and Rangel (2004), similarly, look at the behavior of the agent, theorizing a model in which the consumer can find herself in a hot or cold state: By entering the hot mode, the agent tends to behave instinctually, consuming goods that might reduce, rather than increase, her wealth (Bernheim and Rangel, 2004). The theoretical model has found a compatible match in the perceptions of desire where hot/cold states might compromise self-control efforts of people (Ruttan and Nordgren, 2015).

Hence, dynamic inconsistency can be related to underestimating arousal effects; as explained by Loewenstein (1996) with the hot–cold empathy gap and by Thaler and Sunstein (2009), “when in a cold state, we do not appreciate how much our desires and our behavior will be altered when we are ‘under the influence’ of arousal.” In Loewenstein's model, therefore, the controlled system tries to promote long-term welfare while dealing with the feelings, mischief, and strong will of the hot automatic processes exposed to the temptations that come with arousal (Thaler and Sunstein, 2009). Thus, it emerges the importance of self-control to regulate the emotions of the individual toward the right feeling, which Hoch and Loewenstein (1991) refer to as “consisting efforts on the part of the agent (planner) to avoid or resist behaving in such an inconsistent manner,” affirming self-control mechanisms to be no more than the automatic answers that systemically block tempting behavior.

Control mechanisms stemming from the cooperation between the cognitive and the emotional processes stand within the hot and cold empathy gap (Loewenstein, 1996), driven by a strong motivation and willpower effort, and consume the limited energy necessary to overcome temptation, in the process of ego depletion (Baumeister et al., 1998; Tice et al., 2007; Vohs et al., submitted).

Thaler and Shefrin (1981) identified two control mechanisms that the agents could use to implement self-control and orient their choices: “obscure or avoid, the hot state's impulses deriving from external or internal stimuli, to stay in the cold state” (Metcalfe and Mischel, 1999). The first control mechanism relies on altering the system of incentives by directly modifying the preferences of agents, implementing adequate control mechanisms, or explicitly changing the same incentives (these can be rewards or punishments). The second control mechanism imposes rules reducing the discretion, as a process, either external or internal. The external rules are generally less available and more expensive than the internal ones, which configure as auto-imposed behavioral norms. Internal rules can be chosen freely or learned by other individuals (like family, teachers, and others) and tend to become habits; to be effective, they also need to be simple, have scarce and well-defined exceptions, and be dynamically stable.^3^ Baumeister et al. (2007), in a similar way, explained how commitment to rules, monitoring of appropriate behavior, and the capacity for overriding responses and altering behavior were the three essential components of self-regulation. Bénabou and Pycia (2002) describe self-control as an instrument to avoid the actor questioning the choices she pre-committed by programming and implementing effective control measures.

Several studies have questioned the ways individuals can train self-control to increase concentration in those actions deemed necessary to strengthen self-regulation to follow the preferred path consistently (Metcalfe and Mischel, 1999; Casey et al., 2011). Consequently, self-regulation blocks an immediate sense of satisfaction, allowing the actor to stick to the long-term plan. Bandura (1991) built a self-regulatory process based on setting personal standards and goals: self-observation of actions, self-judgment, and reaction to correct wrong behaviors toward reaching the aim. Muraven et al. (1999) demonstrated that self-control could even be trained through regular practice on small tasks framed by the perceived difficulty, intended as the experience necessary to carry out that task. One could leave those wrong intrinsic mechanisms and develop self-control by inhibiting moods, thoughts, feelings, and urges.

Regarding the field of research studying insurance decision-making, the authors are not aware of any application of the dual-self model or the hot and cold state. We have been able to find some marginal evidence only in a single work, which reported decision-making processes in the insurance sector by theorizing a model that conceives the simultaneous presence of two distinct rational and emotional processes (Bracha and Brown, 2012). In their working paper, the two scholars underline how such processes continuously interact in an intrapersonal game and how the choices are configured due to a Nash equilibrium.

Our choice-making model, presented herein as energy levels, differs from Bracha and Brown's theorized model and aims to evaluate the level of optimal self-control necessary to be exerted to remain in the disciplined cold state.

## Prospect Theory and the Hot–Cold Doers' Interaction

Several models presented up today have no practical validity, as they do not help comprehend the processual phases that can instead be examined using different keys of understanding. The Prospect Theory offers such a practical approach, allowing us to distinguish between rational and irrational decision-makers into the probability weighting. For this reason, we begin our analysis from Kahneman and Tversky's basic equation (Kahneman and Tversky, 1979).

At the decision stage, the agent and consumer can choose between two different lotteries, corresponding to the alternatives “get insurance” and “do not get insurance.” The agent will tend to select that prospect which, in her estimate, has the higher expected value by broadly estimating the probabilities of occurrence and weight for the two outcomes relative to the insurance plan.

The following equation gives the value of the two prospects in the renowned Prospect Theory (Kahneman and Tversky, 1979):

$$\begin{array}{l} V\left(x,p;y,q\right)=\pi \left(p\right)v\left(x\right)+\pi \left(q\;\right)v\left(y\right), \end{array}$$

where *x* and *y* are two possible future outcomes, *p*, and *q*, respectively, their probabilities, *v*(*x*) and *v*(*y*) the utilities, mainly in terms of gain/loss trade-off, of the two outcomes occurring, and π the relative decisional weight. If *p* + *q* = 1 and *x* > *y* > 0 or *x* < *y* < 0, we will have:

$$\begin{array}{l} V\left(x,p;y,q\right)=v\left(y\right)+\pi \left(p\right)\left[v\left(x\right)-v\left(y\;\right)\right]. \end{array}$$

Since the utilities of outcomes, x and y, can be determined in advance and for an almost exact amount, it is evident how estimating the probabilities, other than the decisional weight configure as a strictly subjective process. During the decision phase, the agent experiments almost absolute freedom of estimation, crucially influencing the entire decision-making process. For example, suppose we consider constant the monetary loss relative to the negative outcome *v*(*x*) and the premium to be paid *v*(*y*) and omit π from the analysis the values of the prospects, the future choice will depend solely on the estimation of *p* and *q*. However, the quantification of such probabilities is the harbinger of numerous problems since, far from being executed uniquely through processes that verge to rationality, it is strongly influenced by emotional variables, previous experiences, contingent situations, surrounding environment, and irrational assumptions. The complexity of the problems, the high number of variables to consider, the frequent presence of time and information limitation, and the tendency of individuals to be overconfident toward their evaluations tempt the decision-maker to favor intuitive and irrational approaches (Kahneman, 2011). This stands at the origin of many evaluation errors, which often bring to wrong choices, giving birth to behaviors that, retrospectively, might appear incomprehensible and difficult to explain rationally.

There is an urgent need to adequately dive into the processes through which the agent quantifies such probabilities and chooses the best alternative. Therefore, the dual-self model and prospect theory are particularly effective for this purpose, theorizing an individual composed of a rational and deductive side and an unconscious and impulsive one, with such human components co-existing and conflicting. Adopting such a model facilitates the conceptualization of an agent animated by two sets of opposing preferences. It explains how the same subject can decide in an opposed manner for the same problem. Likewise, in the insurance field, the choices of the consumer seem to arise directly from the conflictual relationship between the described components and the prevalence of one rather than the other.

Hence, we examine the behavior of the consumer in the hot as much as in the cold state. We hypothesize it is the high-arousal state to prevail over the rational one guiding the decision journey in the hot state. In contrast, in the cold state, the decisions of the individuals are more deliberate and taken in a stretched timeframe.

The application of the dual-self model and its contextualization into the hot and cold states, even though surprisingly underused in the research studying the insurance decision-making, allow us to shed some light on those dynamics contributing to understanding the insurance demand. Moreover, adopting the just described approach might also shed light on the regulatory focus theory (Higgins, 1997) crucial in the decision-making process.

When involved in the insurance decision, the perfectly rational choice maker is not identifiable in any choice. Hence, the emotionally affected cognitive state attributable to the so-called doer, the actor of the decision-making instant, constitutes the only utility-consumer of choice, with only one combination systematically prevailing over the others. Which combination prevails will depend on the prevalent hot–cold state and promotion-prevention regulatory focus felt by the individual during the decision moment (Table 1).

**Table 1:** Scenarios of the relationship between hot–cold states and promotion/prevention-focus in emotionally affected cognitive states.

**Regulatory focus**	**State**	
	**Cold**	**Hot**
Promotion focus	Low arousal-positive emotions seeking	High arousal-positive emotions seeking
Prevention focus	Low-arousal negative emotions avoidance	High-arousal negative emotions avoidance

The result of this interaction, a mix of energy utilization/consumption and self-control channelization culminating in the decision-making, will determine whether the agent considers the action consumed as fair, which would allow her to derive utility for the long-term plan.

Let us consider *n* energy states in which the agent consumes utility away from the perfectly rational condition that ideally derives long-term utility. The energy levels related to the two extremes—hot and cold states—result in three possible hypotheses of outcomes based on the positioning of the energy consumption:

*Hypothesis 1*: In the first proposed scenario, the cold state prevails over the hot state. The agent follows a slow decision-making process allowing her for a deliberate decision. Even though the rationality of the decision remains subjective to the decision-makers and their experiences toward the choice, this hypothesis might be configured as the one getting nearer to the ideal long-term utility plan, both in the prevention and in the promotion focus case.

*Hypothesis 2*: The second scenario sees the push for the decision from the hot state being greater than the energy consumed by the cold state. The decision will be made following instinctual heuristics that could deviate from the long-term utility spectrum if not causally related to the decision, hence falling into the category of highly probable “wrong decision.”

*Hypothesis 3*: The third hypothesis equals the amount of energy consumed by the cold state to overcome the temptation to the power exerted by the hot state to follow the instinctual action. In this case, too, the actual fairness of the decision will depend on the heuristics used by the agent and their causal relationship with the object of the decision. The choice's perceived fairness will instead be determined by whether the latter is taken under the prevalent emotions of the agents, making her fall into the desired comfort zone, as is the case for the previous hypothesis.

The following sections describe a fundamental model of insurance decision-making based on energy or utility consumption by the hot–cold states.

## A Model of Insurance Decision-Making Under the Hot and Cold Interaction

Considering an individual with a revenue stream *y* = (*y*_1_,*y*_2_,… *y*_*T*_), with *y*_*T*_ = 0, each agent presents an instant utility function *Z*_*t*_(·), increasing and concaving in *c*_*t*_, while, the long-term utility function *V* = (*Z*_1_, *Z*_2_, …, *Z*_*t*_) is hence represented by the set of actions made in the decision moments. In the rational state, the agent will maximize V by respecting the budget constraint, thus imposing $\sum_{t=1}^{T}{\;c}_{t}\leq \;\sum_{t=1}^{T}\;y_{t}=Y$ (Thaler and Shefrin, 1981).^4^ The instant utility function in the hot state, other than depending on c_*t*_, relies on a modification parameter θ_*t*_, aimed to obtain the desired level c_*t*_ by altering Z_*t*_ in such a way that the perceived utility gradually decreases after hitting a maximum in which ∂Z_*t*_/∂θ_*t*_ < 0. Shefrin and Thaler (1988) gave θ_*t*_ the name of willpower effort variable, specifying how this is the expression of the extent of self-control to bring into play to induce the decision as close as possible to the rationally chosen consumption plan c_*t*_. The use of willpower also implies high cognitive costs (W_*t*_) for which the utility function will, in definitive, be given by

$$\begin{array}{l} Z_{t}=U_{t}+W_{t}\; \end{array}$$

where *U*_*t*_ is associated with the pleasure and positive sensations deriving from consumption and *W*_*t*_ represents the extent of psychologic costs and hence takes a negative value borne, expressed in terms of pain and uncomfortable sensations (Shefrin and Thaler, 1988). Other scholars call such a cost necessary to inhibit impulsive answers as the “cost of attention” (Dukas and Kamil, 2000). Therefore, the perfectly rational agent meticulously evaluates the size of *W*_*t*_ the moment she chooses those control mechanisms that are most effective to orient her actions based on the context. The long-term utility will be derived only if *Z*_*t*_ > 0.

To determine which of the two selves will take the lead for the choice to be made, the decision-making instant is dilated in two different states over time, t_1_ and t_2_, corresponding to a cold state (C) and a hot state (H), respectively. Thus, we will have:

$$\begin{array}{l} Z_{C}=U_{t}+W_{C}\\ Z_{H}=U_{t}+W_{H} \end{array}$$

By comparing the two equations, we have

$$\begin{array}{l} Z_{C}-W_{C}=Z_{H}-W_{H}\; \end{array}$$

Pulling the common terms to the same side of the equation, we observe that:

$$\begin{array}{l} W_{C}-\;W_{H}=Z_{C}-Z_{H}\; \end{array}$$

This model brings us to evaluate the three hypotheses previously considered.

**Figure 1:**
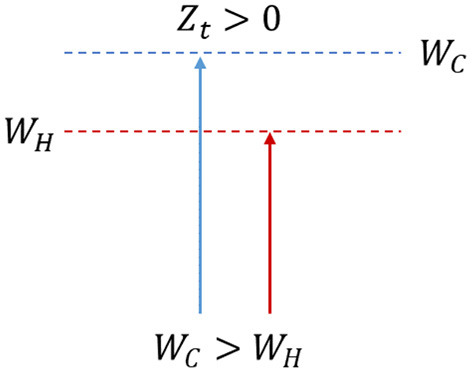
The willpower effort exerted in the cold state is greater than the instinctual response given by the hot state; the individual derives the long-term utility.

*Hypothesis 1*: If *W*_*C*_ > *W*_*H*_ then *Z*_*C*_ > *Z*_*H*_, hence *Z*_*t*_ > 0, the individual derives useful utility, and the decision is aligned with the long-term plan (Figure 1). Thus, this choice will be perceived to be aligned with the chosen path and considered fair in simple terms.

**Figure 2:**
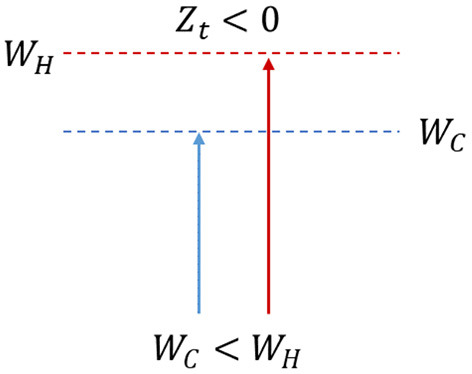
The instinctual hot answer is triggered due to the too high cognitive costs to bear; the conditions to derive utility do not subsist.

*Hypothesis 2*: If *W*_*C*_ < *W*_*H*_, then *Z*_*C*_ < *Z*_*H*_, hence *Z*_*t*_ < 0. The utility consumed by the hot state is not related to the derivation of the long-term utility. With high probability, the agent will be pushed to re-evaluate the long-term plan, thus deviating on a significant level from the original plan, especially in terms of timing (Figure 2).

**Figure 3:**
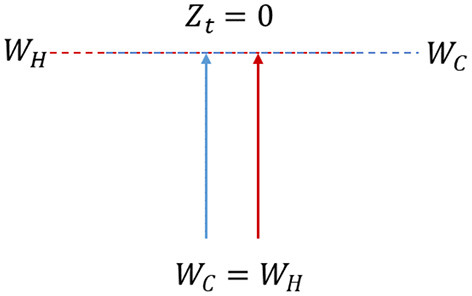
The cold energy exerted equals the hot instinctual answers, thus deriving utility for a 0 amount; the condition for the planner to derive utility does not subsist.

*Hypothesis 3*: If *W*_*C*_ = *W*_*H*_, then *Z*_*C*_ = *Z*_*H*_, hence *Z*_*T*_ = 0. There is no perceived derivation of positive utility, making it very unlikely to make the right insurance decision (Figure 3). The agent will feel regret over the choice due to the incorrect evaluation process.

## Discussion

If the first hypothesis is selected (Figure 1), the agent chooses one among two options in the cold state t_1_: insure (I) or not insure (N). The agent will hence opt for the alternative I if *U*_*CI*_ > *U*_*CN*_ and for N if *U*_*CN*_ ≥ *U*_*CI*_. When the alternatives present the same utility, the agent will choose not to purchase the insurance due mainly to the status quo bias and physiologic aversion to offering (Kahneman et al., 1991). Furthermore, in the cold state, as opposed to the hot states, the overall utility of each of the two alternatives coincides with the benefits deriving from each of these (*U*_*CI*_ = *V*_*CI*_ and *U*_*CN*_ = *V*_*CN*_). During this phase, the deliberate plan, other than guiding the choice of the agent, assumes that her choice could be changed during the following hot states and detects the control mechanisms necessary to avoid it. This is achieved by determining the cognitive costs *W*_*C*_ needed when opting for the other alternative. In this framework, the prevention-focused individual will consider those cognitive mechanisms necessary to avoid the negative emotions related to not purchasing insurance. For example, the event of the death of the agent leaving her family with no monetary cover pushes her toward the rational assumption that the insurance purchase could represent a fair choice. In a promotion-focused individual, the cognitive costs put into play will be those necessary to make her feel the positive emotions related to purchasing the insurance product. For example, even though such a purchase is not necessarily useful, it still provides the agent and the relatives with a financial life-saver. Contrarily, both the prevention and promotion-focused individuals could convince themselves not to insure given the economic burden that would cause them to give up other purchases considered more useful at the moment of the decision.

If the second and third hypotheses are selected (Figures 2, 3), the hot state prevails over or equals the cold one and decisively influences the decision moment t_2_. Thus, in the context of this study, hot states can be categorized into two distinct groups: those that induce the consumer to choose to insure emotionally (t_2I_) and those that induce him not to do so (t_2N_) (Table 2).

**Table 2:** Combinations of possibilities in terms of decision-making under the cold and hot states.

**Decision**	**State**	
	**Cold**	**Hot**
Insure	t_1I_	t_2I_
Do not insure	t_1N_	t_2N_

Consider the most common case, namely, the one in which the agent chooses not to insure in period t_1_ (*U*_*N*_ ≥ *U*_*I*_). After this choice, she can find herself in one of the two previously described hot states and change her mind.

In t_2N_, she will undoubtedly confirm the decision chosen in t_1_, which will appear legitimized by the new situation and will be considered correct even after the intuitive, emotional processes that drive the decision. In t_2N_, the utility deriving from the choice of alternative I will tend not to change to t_1_ or, at most, to be reduced (*U*_*HI*_ = *V*_*HI*_ ≤ *U*_*CI*_ = *V*_*CI*_); on the contrary, the advantages arising from the alternative N will appear as higher if compared to t_1_ and, hence, also the utility deriving from its choice (*U*_*HN*_ = *V*_*HN*_ > *U*_*CN*_ = *V*_*CN*_). From here

$$\begin{array}{l} U_{HN}>U_{HI}\; \end{array}$$

This explains how in t_2N_, the agent will always confirm the cold decision, choosing not to insure. However, in the case of a promotion-focused decision-maker, the hot state will arise in line with the positive emotion-seeking (Higgins, 1997), for example, in the form of an opportunity cost for which the agent could prefer to spend money differently. In this case, it is hypothetically more likely that they will tend not to insure in the hot state.

In t_2I_, the decision-making process appears, on the other hand, more complex. The utility deriving from alternative N will remain constant to t_1_ or will be reduced (*U*_*HN*_ = *V*_*HN*_ ≤ *U*_*CN*_ = *V*_*CN*_). To simplify the exposition, assume that *U*_*HN*_ = *U*_*CN*_. In alternative t_2I_, the resulting benefits will undoubtedly be perceived greater by the emotionally affected “doer” (*V*_*HA*_ > *V*_*CA*_*)*. The utility arising from option t_2I_ will not coincide with the perceived advantages deriving from it but will also account for W's costs that the agent will have to incur to change the decision made in t_1_. We will therefore have that

$$\begin{array}{l} U_{HI}=V_{HI}-W\; \end{array}$$

Also, in such a situation, to make the agent choose the t_2I_ alternative, it will be necessary that

$$\begin{array}{l} U_{HI}>U_{HN}\; \end{array}$$

that is

$$\begin{array}{l} V_{HI}-W>U_{HN}=U_{CN}\; \end{array}$$

Note that *V*_*HI*_ = *V*_*CI*_ + Δ*V*_*HCI*_, where Δ*V*_*HCI*_ represents the variation of the perceived advantages in t_2_ compared to t_1._ The agent in the hot state will hence tend to choose the alternative I if

$$\begin{array}{l} \Delta V_{HCI}>\left(V_{CN}-V_{CI}\right)+W\; \end{array}$$

the variation of benefits deriving from purchasing the insurance will be greater than the sum between the cognitive costs to bear for the change of choice and the difference between the advantages deriving from the alternatives N and I in t_1_. Since the agent acquires as given *V*_*CN*_, *V*_*CI*_ and *W* (determined by the rational choice of the agent in t_1_), her decision will depend almost exclusively on her estimate of the advantages deriving from the purchase of the insurance and, more specifically, how far it will deviate (positively) from that made originally. If that is the case, the impulsive evaluation made by the hot state will prevail. On the other hand, prevention-focused individuals will be driven by heuristics relative to safety, responsibility, and willingness to avoid mistakes. In this case, the agent will hypothetically overvalue the risks of adverse events and be more likely to purchase insurance in such a scenario.

Thus, the heuristics which bring the agent toward the hot state have not been effectively fought by the control mechanisms implemented to maintain the decision moment in a cold state. In other words, the amount of willpower utilized has not been significant enough to graduate the push of arousal. For these reasons, the decision-making in the second and third hypotheses cannot be configured of granting a fair choice and the feeling of a positive derivation of utility to the deliberate and forward-looking system.

However, to overcome promotion- and prevention-focused concerns, individuals can still utilize willpower effort on their self-control to overcome the ideal-self-related heuristics toward the unaffected choice that most respects the long-term utility.

## Conclusions

It is reasonable to ask, at this point, which state presides the decision-making process majorly, orienting the choice toward which one of the other alternatives. The heuristics of availability, accessibility, and representativeness, which play a crucial role in the described process, significantly affect the decision-maker.

The uncertainty relative to the insurance purchase decision places it under the category of those not best suited for a rational judgment with an exact answer; hence, the traditional conflict between cognition and emotion materializes even during this process. Therefore, the self-control mechanisms have a minor magnitude but are not absent. It is always the reflective system to grant intuitive and approximate answers that have an appearance of objectivity; this interaction becomes particularly effective primarily by adopting rules of interpretation and evaluation that are necessarily set and evaluated *ex ante*. These rules should prevent the agent from making gross evaluation mistakes, especially in the so-called hot states. The effective set of incentives positively alters the exertion of willpower necessary to train self-regulation and strengthen resistance to the cognitive costs. It is also of tremendous importance to place the agents in a healthy environment that positively influences the right direction. Further, it is crucial to ensure a regulated competitive sector to maximize the trust of the agent in the insurance company.

In some cases, however, even though self-control might be an excellent instrument to direct the consumer nearer to the rational decision, uncertainty still holds. The reason for which the agent moves the center of the decisional process from the calculation and evaluation of probabilities to a mere review of the price of the insurance: Banally, he will compare the (maximum) price he is willing to pay with the price set by the offer. This evaluation is fostered by the algorithmic matching made by the insurance company when the data of customers are inserted. When the difference between the expected price and the effective one is positive, the consumer will be more incentivized to purchase the product because of the value of what Thaler (2018) defines as “transaction utility.”^5^

When the transaction utility is negative, contrarily, the consumer will be less propense to the purchase. Issues arise, however, since transaction utility can differ, sometimes to a great extent, from the purchasing utility. It coincides with what is commonly called in economics “the consumer's surplus,” alias the difference between the utility obtained (or obtainable in case of insurance) with the purchase of the selected product and the cost opportunity of the alternatives (Thaler, 2018). In a decisional process governed by the planner and centered on rationality and objectivity, the agent should consider only the purchase utility in his decisions. However, in complex situations, the choice is reduced systemically to an evaluation exclusively based on transaction utility, falsely granting the decision-maker the feeling of having made the right decision.

In addition to the objective reasoning in purchase utility presented before, a typical application of the model presented herein uses behavioral distortions in leading to changes in insurance demand. The representative heuristic has been shown to increase aggregate demand for insurance in the presence of recent losses and lower demand when a loss has not occurred recently. At the same time, similarly, availability bias captures the ease with which the association of an event can be brought to mind (Dumm et al., 2020). Now, let us consider the classic example of a person who rationally believed in the cold state (t_1N_) to need no insurance, for instance, against credit card theft at an effective price considered too high since he did not experience such a burden before. Later, his credit card data were stolen. The agent started seeing the loss of the data and potential fraud as an action he could have avoided earlier, reflecting this fear by tending to erroneously overestimate the probability of that event to occur again in the future. Driven by the strong impulse to remedy this possibility and the recent availability, represented in Table 1 as high-arousal negative emotions avoidance, he reconsiders the transactional utility derived by the optimal expected price that will fall above the decision's effective price in t_2I_. At this point, the agent will be led to purchase the insurance that would have been otherwise refused. The same conclusion could arise if the individual tries to compensate for the loss by actualizing the expected refund after the insurance subscription and another theft. Even in this case, this would not happen if the choice had been left to the rational decision and the implemented control mechanisms were effective. The planner would be able to fight the representative inductions of the doer and settle the choice on more reliable criteria. For this reason, the insurance company should act as a cushion for the agent in the hot state not to make that mistake, maybe adding to the algorithmic evaluation a question regarding similar events to the specific insurance that may have recently happened to the potential customer. Other than for the agent herself, this may be useful to get to a higher rate of customer satisfaction toward the provided consultancy service.

Reformulating the question on a more general level, is it rational or instinctual to determine the choice of the agent toward purchasing the insurance plan? And what is the role of self-control in such a process?

The answers to the questions are not unique. What we said so far could lead to the erroneous conclusion that it is the automatic system that positively influences the choice. Indeed, system one is responsible for the action itself. Still, at the same time, system two is reliable in directing the decision of the instinctual self toward the choice nearest to the utility maximization. This happens mainly during the cold state when the information is greater, there are few external stimuli, and the emotional variables do not spend significant amounts of energy on the person. Repeated actions correctly utilize self-control and form positive heuristics that systematically drive the decision-maker toward decisions perceived as reasonable and fair. Instead, as highlighted in the example of the credit card theft provided in the hot state, the agent is more inclined to implement heuristics and conduct choices based on subjective, erroneous, and transitory evaluations. During such moments, the control mechanisms should ease the blind action and attitude of the agent to maximize instantaneous utility.

It becomes necessary to reflect on the relationship between good and bad and ask ourselves, concerning the argument of analysis of this work, if the lifetime utility maximization of the agent represents the highest good for the agent and what the individual considered in her entirety and desires. We may hypothesize the role of self-control as an intermediary to balance up the long-term needs with the irrational instincts, to give the agent a sense of immediate satisfaction that helps in the long term the agent to thrive for the goals set by the planner.

## Limitations

Different areas of investigation of the mechanism underneath the decision-making in conditions of uncertainty have not agreed on a shared model to explain the way choices are made. The present article proposes to mark a starting point of gathering between behavioral economic theory of utility maximization and cognitive psychology in the insurance decision-making process. Although, the interdisciplinarity between the economic field and the psychological one poses some challenges given the previous attrition from the classical economic theories (Samuelson, 1937). Modern models have been crucial to highlight the systematic contradictions from the ideally rational agent (Strotz, 1955; Ainslie, 1975; Thaler and Shefrin, 1981) toward the psychological theories of decision-making (Loewenstein, 1996; Higgins, 1997; Bernheim and Rangel, 2004). It would be recommended to carefully evaluate combinations of those theories in empirical and ecological conditions under a common investigation term. As reported by Glimcher (2015), “decision-making systems were trading off representational costs at the neurobiological level against benefits at the level of behavior—and that this trade-off might account for the apparent gap between the *whys* of economists and the *whats* of psychologists”.

The diversity of the choices plays a role in providing different opinions, and contextualizing in the life insurance field might furnish interesting findings that are difficult to translate into other decision-making spheres. The different characteristics of the individuals, intended as the result of their experiences, also provide the study of decision-making of consumers with a set of *n* personalities that emotionally react in different directions to certain impulses. The theoretical model reported in this work could be confuted or confirmed by experimental research for this purpose.

## Future Research

It is now clear that environmental, social, and cultural conditions that should not be included in the evaluation process are instead a part of it due to the inner characteristics of individuals finding themselves to choose in the presence of uncertainty. These characteristics would provide a probability to perceive the value of an insurance product as incredibly useful at a specific moment while useless at a second time. The decision in such a scenario assumes casual characteristics for which the actual utility of the purchase often does not coincide with the ideal one. For example, the inception of the COVID-19 pandemic and the relative lockdown forced citizens home, with a global threat for health safety that has caused interruptions in the macroeconomic environment and shaken the foundations of health governance all over the world (Cori et al., 2020). The lockdown and social distancing to combat the COVID-19 virus have generated significant disruptions in consumer behavior due to modifying risk perceptions of individuals (Sheth, 2020). Subjectivity in probability weighting of rare events, as in the current COVID-19 pandemic, and representativeness heuristic (Berenbaum, 2021) response to the information gap created between the cold and the hot states (Phelps and Pollak, 1968; Blackorby et al., 1973; Peleg and Yaari, 1973; Ainslie, 1975; Hammond, 1976; Frederick et al., 2002).

Future empirical research might be helpful to provide validation or confute the model of the present paper. Neuroscientific studies might be a complementary field of study between psychology and behavioral economics to identify the neural correlates associated with the proposed model. For example, Kang and Camerer (2013) investigated the neural findings associated with hypothetical bias and recorded brain activity to create better forecasts of actual consumer choice. Indeed, subjects pay more to avoid bad decisions when the choice is real, thus inverting the hot–cold empathy gaps in hypothetical conditions. This effect might be a weakness to be considered when creating empirical studies without considering the effective ecological setting. It would be interesting for this study to assess the propensity to seek insurance coverage or not with subjects who have experienced a loss or the infection by COVID-19 of a loved one, correlating the distinct activity in diverse regions of the brain.

This analysis can only be elaborated by taking as a starting point the dual-self model and accounting for the perceived value of a purchase that affects an individual in the two main directions of promotion-focus vs. prevention-focus. Furthermore, an empirical analysis could also help classify the conditions under which these behaviors are present and how positive emotion seeking and negative emotions avoid influencing insurance decision-making. Finally, such a classification might be helpful for institutional policymakers to center the decisions of the majority toward a utility-maximizing choice made in the best hot–cold state possible for the customer not to look behind.

## Author Contributions

AM: conceptualization, funding acquisition, supervision, validation, and writing—review and editing. MA: methodology and writing—original draft. All authors contributed to the article and approved the submitted version.

## Conflict of Interest

The authors declare that the research was conducted in the absence of any commercial or financial relationships that could be construed as a potential conflict of interest.

## Publisher's Note

All claims expressed in this article are solely those of the authors and do not necessarily represent those of their affiliated organizations, or those of the publisher, the editors and the reviewers. Any product that may be evaluated in this article, or claim that may be made by its manufacturer, is not guaranteed or endorsed by the publisher.

## Footnotes

^1^Ut (ct,…, cT) = $\overset{T-t}{\underset{K=0}{\sum }}\;D\left(k\right)u\left(c_{t+k}\right)$, where *D*(*k*) = δ^*k*^ and *u*(·) represent the instantaneous utility function, indicating the preferences of the agent for the consumption of *c*_*t*_; δ, with a value of less than 1, is the intertemporal discount factor, that is, the parameter through which future consumption is evaluated.

^2^Thaler and Shefrin (1981) especially refers to Pollak (1968), Blackorby et al. (1973), Hammond (1976), and Peleg and Yaari (1973).

^3^In their definition of optimal characteristics of internal rules, Shefrin and Thaler resume literature on impulse control, particularly referring to Ainslie (1975).

^4^In Shefrin and Thaler (1988), Y is indicated as LW, standing for Lifetime Wealth.

^5^*p* = Expected price. p^*^ = Effective price. We will then call TU the customer's transaction utility, given by the following difference: TU = p°-p^*^.
